# Understanding bias when estimating life expectancy from age at death: a simulation approach applied to Morquio syndrome A

**DOI:** 10.1186/s13104-021-05894-0

**Published:** 2022-01-15

**Authors:** Xue Yin, Jaeil Ahn, Simina M. Boca

**Affiliations:** 1 Department of Biostatistics, Bioinformatics and Biomathematics, Georgetown University Medical Center, Washington, DC USA; 2 Department of Oncology, Georgetown University Medical Center, Washington, DC USA; 3 Innovation Center for Biomedical Informatics, Georgetown University Medical Center, Washington, DC USA; 4 Present Address: Medpace, Cincinnati, OH USA; 5 Present Address: AstraZeneca, Gaithersburg, MD USA

**Keywords:** Life expectancy, Morquio syndrome A, Simulations, Kaplan–Meier

## Abstract

**Objective:**

Life expectancy can be estimated accurately from a cohort of individuals born in the same year and followed from birth to death. However, due to the resource-consuming nature of following a cohort prospectively, life expectancy is often assessed based upon retrospective death record reviews. This conventional approach may lead to potentially biased estimates, in particular when estimating life expectancy of rare diseases such as Morquio syndrome A. We investigated the accuracy of life expectancy estimation using death records by simulating the survival of individuals with Morquio syndrome A under four different scenarios.

**Results:**

When life expectancy was constant during the entire period, using death data did not result in a biased estimate. However, when life expectancy increased over time, as is often expected to be the case in rare diseases, using only death data led to a substantial underestimation of life expectancy. We emphasize that it is therefore crucial to understand how estimates of life expectancy are obtained, to interpret them in an appropriate context, and to assess estimation methods within a sensitivity analysis framework, similar to the simulations performed herein.

**Supplementary Information:**

The online version contains supplementary material available at 10.1186/s13104-021-05894-0.

## Introduction

Life expectancy is generally defined as the amount of time an individual can expect to live from birth; thus, it may refer to either the mean life expectancy or the median life expectancy. There are two main approaches to estimating life expectancy: cohort and period life expectancy. Cohort life expectancy is the average length of life from an actual cohort of individuals born in the same period. Since it is challenging to follow up individuals from birth to death prospectively, life expectancy is often evaluated using the average length of life in a hypothetical cohort of individuals who are assumed to have been born and died with the mortality rate observed in the same period [1, 2], known as period life expectancy.

The use of period life expectancy may result in a biased estimate if the true life expectancy changes over time. For instance, it will exclude living individuals, meaning that any recent changes in survival, including potential increases due to improved diets, environmental changes, or biomedical innovation [3], will not be accounted for [4]. This problem is exacerbated in the case of life-limiting, rare genetic diseases such as Morquio syndrome A (also known as mucopolysaccharidosis type IVA or MPS IVA), a Mendelian autosomal recessive disorder [5] which has an estimated birth prevalence (incidence) between 1 in 71,000 and 1 in 179,000 [5]. For such diseases, it will be more difficult to identify a sufficient number of individuals to follow from birth to death. With limited data, the estimation can heavily rely on assumptions that may not fully reflect contemporary situations. For instance, given the potentially impactful benefits of early diagnosis and improvements in general care [6], the life expectancy of individuals with rare diseases may be more likely to have increased in recent years compared to the life expectancy of the general population.

Life expectancy for individuals with MPS IVA was previously estimated based on a total of 27 deaths that occurred over 36 years—between 1975 and 2010—in the United Kingdom [7]. The mean age at death was 25.3 years (range of 3.08–75.32 years). We first tried to reproduce the analysis in [7] that estimated the association between age at death and year of death using the data provided therein and obtain similar, but not identical results (see Additional file 1).

In the current study, we consider a simulation approach to investigate how using data on only deceased individuals—in other words, the period approach—as in [7], can lead to biases when estimating life expectancy. As these estimates may be used by individuals with MPS IVA and their caregivers and providers to understand disease prognosis and plan possible interventions, it is crucial to provide a more nuanced interpretation and increase understanding of potential biases.

## Main text

### Methods and results

#### Simulation scenarios

We conduct simulation experiments to understand situations when biases in life expectancy estimation arise using four pragmatic scenarios. In all scenarios, we simulate the birth and death of individuals based on the Weibull distribution assuming an annual birth prevalence of one individual with MPS IVA born over 500 years. To mimic the MPS IVA data collection in [7], we focus on the life expectancy for individuals born in the last 36 years of the simulation, discarding the majority of the simulated individuals. We considered 500 years of forward simulation time to ensure that we would obtain a large enough number of deaths in the period [465, 500] to enable us to proceed with our analyses. Individuals born in year 1 have the same mean and median survival as in [7] (Mean=25.3, Median=20.8). We repeated 1000 simulations to obtain summary statistics, with  bias defined as “the true life mean or median expectancy minus the averaged mean or median expectancy from 1000 simulations.” Descriptions of four scenarios are given below, with added details in Additional file 1.

##### Scenario 1: constant life expectancy

We first assume that life expectancy is constant over 500 years. We generate survival from the Weibull distribution (scale parameter $\lambda =27.46$, shape parameter $k=1.32$).

##### Scenario 2: gradually increasing life expectancy

In this scenario, we assume that life expectancy increases linearly over time. This may reflect a situation where the treatment or care for MPS IVA has been consistently improving each year. Here we set the mean and median survival to increase by 0.05 years each year.

##### Scenario 3: gradually increasing life expectancy that later stabilizes

Life expectancy may also improve up to a certain year, then stabilize without further improvements, for example, if a care protocol is refined up to a certain point, then stops improving, but continues being utilized and effective. We model this via a scenario where life expectancy increases for the first 460 years (by 0.05 for mean and median survival), then stabilizes in the last 40 years.

##### Scenario 4: constant, then increasing life expectancy

Finally, we assume that life expectancy is stable for the first 460 years and only increases in the last 40 years, for example, where a new standard of care is established that leads to a gradual improvement. We assume this treatment is more effective than treatments from the second and third scenarios, increasing mean survival by 0.5 years per year starting in the year 460.

#### Methods for estimating life expectancy

We consider a variety of approaches to estimate the life expectancy of individuals who were alive at some point within the last 36 years of the simulated time period. The period life expectancy approach is equivalent to [7], using only data on the individuals who died during that period to estimate mean and median life expectancy. For the cohort approach, we use the full survival times of individuals born between years 465 and 500; however, in practice, we note that death data after year 500 is not feasible if the life expectancy analysis is performed at year 500.

We also consider the Kaplan–Meier (KM) method to estimate the median survival in the presence of censored data [8], which allows us to include partial survival times as censored times for individuals who are still alive at year 500. We estimate median life expectancy for both retrospective (R) sampling, where we consider individuals who died since year 465 and prospective (P) sampling, where we instead follow up individuals who were born since year 465. Estimating the median survival time of all individuals who died between years 465 and 500 without censoring at year 500 via the KM approach is equivalent to the period life expectancy approach. If censoring is considered at year 500, the resulting survival times are often heavily censored. To address this problem, we also consider KM estimation that weights censored individuals and reduces their influence. Specifically, we considered both weighting censored individuals by 0.1—due to this factor working well in practice —and by the percentage of uncensored individuals (uncensored percentage) among all sampled individuals [9]. For prospective sampling, the KM estimation censors all individuals still alive at year 500.

#### Simulation results

**Fig. 1:**
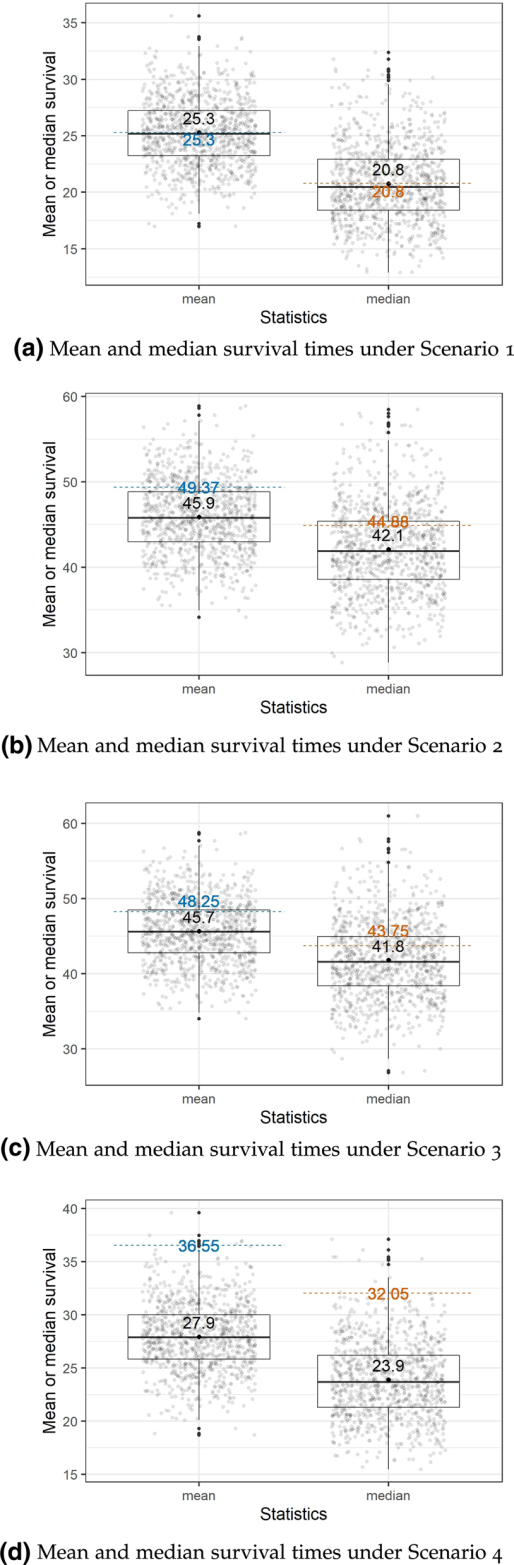
Boxplots of estimated period mean and median survival times for simulation scenarios 1–4. The blue dashed line is the average of the true mean survival times for the last 36 years. The orange dashed line is the average of the true median survival times for the last 36 years. Each grey point represents the result of a single simulation run, with the means and medians estimated via the period approach, using only the simulated individuals who died in the last 36 years within that run. The black point is the average value of the grey points over 1000 simulation runs

Results from the four simulation scenarios—giving the true mean and median life expectancies and the estimates from the period approach —are presented in Table 1 and Fig. 1. Table 1 presents the summary results using the mean period and cohort estimates and Fig. 1 shows the individual results for each of the 1000 simulations runs for the period approach. For scenarios 2–4, where the life expectancy was not constant over the entire period, the “true values” are given by the average mean and median values over the last 36 years.

For the first scenario of constant life expectancy, the estimated period means and medians from the 1000 simulation runs are close to the true values, with no notable bias. However, with the departure from the constant life expectancy assumption in the remaining three scenarios, the estimated period mean and median estimates consistently are underestimated. The estimated cohort mean and median survival estimates—which use all individuals born since the year 465—are close to the true values in all four scenarios.

**Table 1:** True mean and median survival and average estimated mean and median survival across 1000 simulation runs, for each of the 4 simulation scenarios, using both the period and cohort estimation approaches

Scenario	Mean survival time			Median survival time		
	True value	Estimates		True value	Estimates	
		Period	Cohort		Period	Cohort
1	25.30	25.24 (2.92)	25.35 (3.28)	20.80	20.75 (3.68)	20.89 (3.68)
2	49.37	45.91 (4.18)	49.52 (4.95)	44.88	42.10 (6.02)	44.96 (6.02)
3	48.25	45.67 (4.2)	48.37 (4.89)	43.75	41.83 (5.91)	43.77 (5.91)
4	36.55	27.93 (3.16)	36.63 (4.10)	32.05	23.90 (4.99)	31.96 (4.99)

The true values represent averages over the last 36 years for each scenario. The period and cohort approaches average the corresponding estimates over 1000 simulation runs. The numbers in parentheses represent the standard deviations across the 1000 simulation runs

The average KM estimates for the four simulation scenarios are shown in Table 2. Considering the individuals who are still alive at year 500 as censored, the KM approach overestimates the median life expectancy under all scenarios. More details are provided in Additional file 1.

**Table 2:** Average number of deaths in the time period [465, 500] years and Kaplan–Meier estimates of the median survival time over the four simulation scenarios, over 1000 simulation runs in each scenario

	Scenario			
	1	2	3	4
Average number of deaths				
In [465, 500]	34.97	33.39	33.59	30.54
Since 465	60.79	81.27	81.23	60.79
R				
Average KM estimates				
Died in [465, 500] (no censoring)	20.75	42.10	41.83	23.90
Died since 465 (censored at 500)	29.84	60.16	59.95	33.78
Died in [465, 500] (censored at 500, weighted by 0.1)	21.69	44.25	43.98	25.06
Died in [465, 500] (censored at 500, weighted by uncensored percentage)	26.01	50.3	50.1	29.16
P				
Average number of deaths for individuals born since 465				
In [465, 500]	14.41	5.29	5.48	10.06
Since 465	36	36	36	36
Average KM estimates				
Born since 465 (censored at 500)	25.03	NA	NA	27.61

*R* retrospective sampling (considering individuals who died since a certain year), *P* prospective sampling (considering individuals who were born after a certain year), *NA* estimation not possible due to small number of events

### Conclusions and discussion

We investigated the problem of estimating life expectancy using data on age at death (the period approach), focusing on Morquio syndrome A [7] by simulating four scenarios to better understand the magnitude and types of biases that may occur. When life expectancy was constant, this method performed well, lacking bias. This is known to be the case under constant mortality rates [4]. However, this scenario fails to reflect the advances in medical practices and technology. Changes in general care and treatment of MPS IVA—such as enzyme replacement therapy and hematopoietic stem cell therapy [10]—are expected to lead to a possible rise in life expectancy [7]. Even in the absence of specific treatments, standards of care have improved along with advancements in medical devices and techniques [11]. In all the non-constant life expectancy scenarios we considered, which represented a mix of stable and increasing life expectancy, period life expectancy substantially underestimated both the mean and the median life expectancy. Thus, unless the life expectancy is relatively stable over time, the period approach used in [7] will not accurately assess life expectancy.

In contrast with period life expectancy, which assumes stable mortality rates over the period of interest, cohort life expectancy allows for possibly time-varying mortality rates, the two being equivalent when mortality is constant [4]. When life expectancy increases, the period approach underestimates the true life expectancy, while the cohort approach leads to unbiased estimates. However, the cohort approach is generally impractical, especially for rare diseases like MPS IVA, which make it more challenging to find and follow up a large enough number of individuals. Moreover, with a cohort approach, there is always a lag between the life expectancy estimated on a cohort for which everyone is deceased and the life expectancy for an individual born at the present time.

We also considered the KM method—which allows for the inclusion of individuals who are still alive as “censored data”—to estimate the median survival, which did not eliminate bias, although it changed its direction. A more detailed discussion can be found in Additional file 1.

## Limitations

Our study’s most important limitation is that the simulated datasets are based on simplified assumptions, as there is insufficient knowledge of changes in the natural history of Morquio syndrome over time to build more detailed models. This led to our choice of a number of scenarios as a de facto sensitivity analysis for comparing the performance of various approaches to estimate life expectancy. We also note that while in general, life expectancy is expected to increase over time, this trend is sometimes unstable and even unpredictable. For instance, the current COVID-19 pandemic may lead to a reduced lifespan for individuals with underlying respiratory conditions [12], including MPS IVA. To estimate the life expectancy more accurately, we need to consider contemporary changes in the standard of care and medical treatment and the possibility of unforeseeable and unfavorable events, which may include pandemics.

When estimating life expectancy, especially in rare diseases, using the cohort approach with long-term data will be more accurate than using the period approach, but this design also has drawbacks and results in lagged estimates. If using the period approach, we must assume that the result will not reflect the current life expectancy and will probably underestimate it in the case of improvements in treatment or general care. An alternative is to also include data on individuals who are still alive by employing a weighted KM method, though this appears to lead to anti-conservative biases and strongly depends on the chosen weights. Future methods will require a balance of these aspects, potentially by incorporating certain reasonable assumptions on the underlying life expectancy and considering extensive sensitivity analyses.

## Supplementary Information

**Additional file 1. **Supplementary information to “Understanding bias when estimating life expectancy from age at death: A simulation approach applied to Morquio Syndrome A”.

## Data Availability

All the analyses in this manuscript are reproducible, with the code and data available at https://github.com/xueyin97/Bias-estimating-life-expectancy-Morquio.

## Abbreviations

MPS IV: Morquio syndrome A (also known as also known as mucopolysaccharidosis type IVA)
KM: Kaplan–Meier approach
R: Retrospective
P: Prospective
