# Supplementary material for: Understanding bias when estimating life expectancy from age at death: a simulation approach applied to Morquio syndrome A
Source: BMC Res Notes. 2022 Jan 15;15:19. doi: 10.1186/s13104-021-05894-0 (PMC8760562; doi:10.1186/s13104-021-05894-0)
Supplement: Supplementary file 1 — Additional file 1. Supplementary information to “Understanding bias when estimating life expectancy from age at death: A simulation approach applied to Morquio Syndrome A”. [file 13104_2021_5894_MOESM1_ESM.pdf]

Supplementary information to *Understanding bias when estimating life expectancy from age at death: A simulation approach applied to Morquio Syndrome A*

January 6, 2022

REPRODUCING THE LAVERY 2014 ANALYSIS

We first attempted to reproduce the analysis in [1] by fitting a simple linear regression model. We used age at death (y) against the year of death (x), considering changes in the life expectancy of individuals with MPS IVA from 1975 to 2010. This analysis showed that affected individuals had a slow linear growth over time in life expectancy, $y = -1424.9 + 0.7368x$ ($R^2 = 0.0963$). Using the data provided in [1], we obtained a similar, but not identical result: $y = -1344.7209 + 0.6862x$ ($R^2 = 0.0922$). While there is a slight discrepancy between the data presented in [1] and the data used by those authors to fit the linear model, we do not anticipate this to be a major issue.

ADDITIONAL DETAILS FOR SIMULATION SCENARIOS

Scenario 1: Constant life expectancy

The first scenario, shown in Figure 1a, concerns a less realistic situation where the life expectancy is constant over 500 years. This can occur when there is no advancement in treatment or no change in environment that will influence the life expectancy of individuals with MPS IVA during the entire 500 year period. We generate survival from the Weibull distribution with scale parameter $\lambda = 27.46$ and shape

parameter $k = 1.32$, which are obtained by using the mean of 25.3 years and median of 20.8 in the equations:

$$Mean = \lambda\Gamma(1 + \frac{1}{k}), \qquad Median = \lambda(ln2)^{\frac{1}{k}},$$

where Γ is the gamma function, and solving for λ and k. The density of this distribution is shown in Figure 2.

Scenario 2: Gradually increasing life expectancy

Contrary to the first simulation scenario with constant life expectancy, we assume that life expectancy increases linearly over time as in Figure 1b. This may reflect a situation where the treatment or care for MPS IVA has been consistently improving each year. This situation can occur when the standard of care gradually improves over time, without the introduction of a state-of-the-art intervention that suddenly replaces the existing standard of care. Here we set the mean and median survival to increase by 0.05 years each year. In this case, a simulated individual born in year 410 has the mean and median survival time of 50.25 and 45.75 years, respectively.

Scenario 3: Gradually increasing life expectancy that later stabilizes

An alternative scenario is that life expectancy may improve up to a certain year, then stabilize without further improvements, for example if a treatment or a care protocol is refined up to a certain point, then stops improving, but continues being utilized and effective. For instance, while surgery complications appear to have reduced over time [1], there may be a point past which surgery can no longer further improve outcomes for individuals with MPS IVA. We model this via a scenario where life expectancy increases for the first 460 years, then stabilizes in the last 40 years, as visualized in Figure 1c. The mean and median survival increases by 0.05 years per birth year until year 460. In this case, a simulated individual born in the year 460 has a mean and median survival of 48.25 and 43.75 years, respectively. Life expectancy is then constant until the year 500.

Scenario 4: Constant, then increasing life expectancy

In this scenario, we assume that life expectancy is stable for the first 460 years and only increases in the last 40 years, as shown in Figure 1d. This can be a situation where a new standard of care is established and it leads to a gradual improvement. We assume this treatment is more effective than the treatments from the second and third scenarios, increasing mean survival by 0.5 years per year starting in the year 460, meaning that individuals born in the year 500 have a mean survival time of 45.3 and a median survival time of 40.8 years.

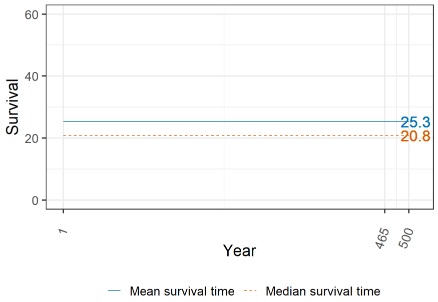

(a) Scenario 1: Mean and median are constant over the entire 500 year period.

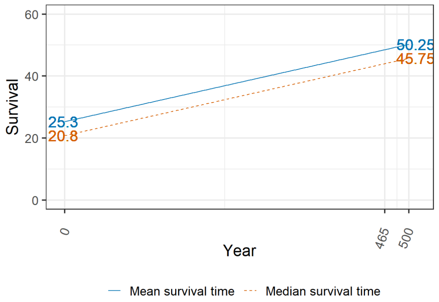

(b) Scenario 2: Mean and median change by increments of 0.05 years per birth year.

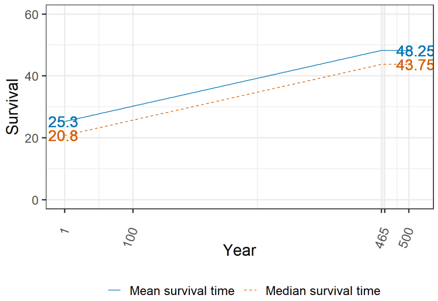

(c) Scenario 3: Mean and median change by increments of 0.05 years per birth year, then stay flat for last 40 years.

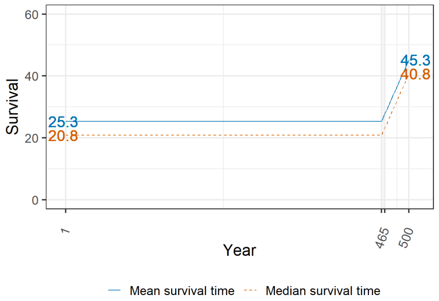

(d) Scenario 4: Mean and median are constant, except for the last 40 years, when they change by increments of 0.5 years per birth year.

Supplemental Figure 1: True mean and median values for the different scenarios. The solid blue line represents the true mean survival time. The orange dashed line represents the true median survival time.

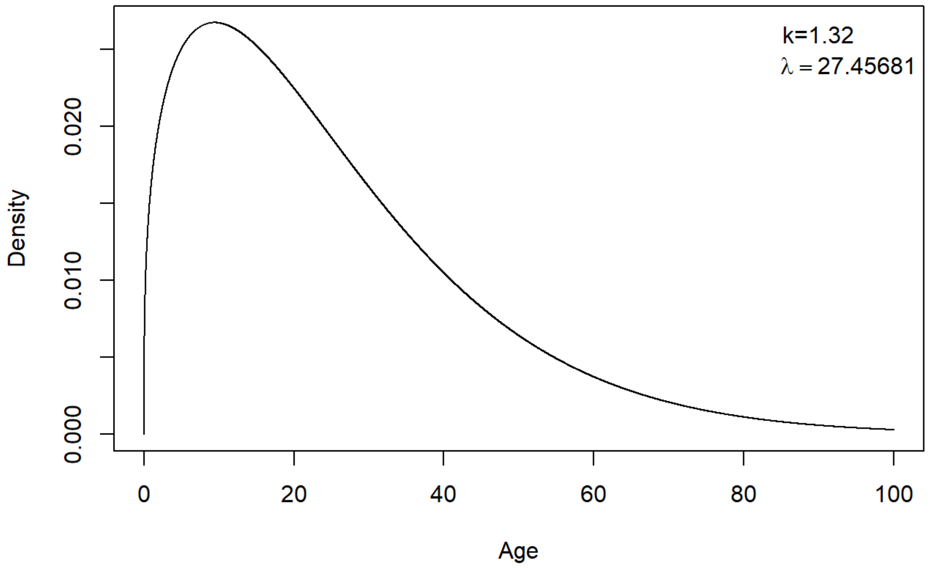

Supplemental Figure 2: The density of the Weibull distribution used for generating survival times for the first simulation scenario, which considers constant mean and median survival of 25.3 and 20.8 years respectively, over a 500 year period.

DETAILED RESULTS FOR KM APPROACHES

The KM estimate using only individuals who died between years 465 and 500 (with no censoring at year 500) is the same as the period life expectancy estimate, but we include it here as well for comparison with the other KM estimates. The KM approach weighting censored individuals by 0.1 — which reduces the influence of heavy censoring — yields median survivals closer to the true medians than the other KM methods. When weighting by the uncensored percentage among those individuals, the KM estimator performs worse than when weighting by 0.1 but is closer to the true median compared to the versions that do not weigh the censored data. For the prospective approach, which considers individuals born since year 465 and censored at year 500, the KM method cannot be fit in Scenarios 2 and 3, because on average, only around 5 individuals die within the period [465, 500] years, the remainder being censored.

DETAILED DISCUSSION FOR KM APPROACHES

The KM estimate for individuals who died between the years 465 and 500, censoring individuals still alive at year 500, overestimated true survival in all four scenarios. This may be expected given the high rate of censoring — on average, 58% of individuals were censored under the first scenario, 41% percent under the second and the third, and 50% for the fourth scenario — and the tendency of the KM estimator to be anti-conservative in these conditions [2, 3]. To overcome this problem, we considered using weights for the censored data. Weighting the KM median by 0.1 leads to greatly improved estimates, but since it was chosen based on the known "ground truth" in simulated examples, this cannot be recommended in general. Approaches also exist to weight the censored data by the percentage of the uncensored individuals among all sampled individuals [4, 3, 5]. This still leads to an overestimation of the median survival, but provides a better option than the unweighted KM method. We also considered a prospective sampling design, following up individuals who were born since year 465, but censoring them at year 500. In this case, since we only have 36 individuals born since 465 due to our assumptions, it becomes impossible to obtain an estimate in two of the scenarios, as the average number of deaths for those individuals in the period [465, 500] years is below 6.

REFERENCES

[1] C. Lavery and C. Hendriksz. Mortality in Patients with Morquio Syndrome A. *JIMD Reports*, 15, 2014.

[2] M. Noordzij, K. Leffondré, K. Stralen, and et al. When do we need competing risks methods for survival analysis in nephrology? *Nephrology Dialysis Transplantation*, 28:2670–2677, 11 2013.

[3] K. Shoaib, A. Hamdi, and A.L. Ahmed. A compression of Kaplan Meier vs. weighted Kaplan-Meier in comparing estimation of heavy censoring data. *American Scientific Research Journal for Engineering, Technology, and Sciences*, 36:211–223, 2017.

[4] M. Shafiq, S. Shah, and M. Alamgir. Modified weighted kaplan-meier estimator. *Pakistan Journal of Statistics and Operation Research*, 3(1)):39–44, 2007.

[5] A. Zare, M. Mahmoodi, K. Mohammad, and et al. A comparison between Kaplan-Meier and weighted Kaplan-Meier methods of

five-year survival estimation of patients with gastric cancer. *Acta Medica Iranica*, 52(10):764–7, 2014.
